# Purification, biochemical characterization, and biotechnological applications of a multifunctional enzyme from the *Thermoascus aurantiacus* PI3S3 strain

**DOI:** 10.1038/s41598-024-55665-7

**Published:** 2024-02-29

**Authors:** Juliane Almeida Battisti, Giovane Bruno Rocha, Letícia Mara Rasbold, Vitória Maciel Delai, Monica Sarolli Silva de Mendonça Costa, Marina Kimiko Kadowaki, José Luis da Conceição Silva, Rita de Cássia Garcia Simão, Thaís Duarte Bifano, Alexandre Maller

**Affiliations:** 1 Centro de Ciências Médicas e Farmacêuticas, Universidade Estadual do Oeste do Paraná, 2069 Universitária Street, Faculdade, Cascavel, Paraná 85819-110 Brazil; 2 Centro de Ciências Exatas e Tecnológicas, Universidade Estadual do Oeste do Paraná, Cascavel, Paraná 85819-110 Brazil

**Keywords:** Fungal enzyme, Promiscuous enzyme, Glycoside hydrolase, Juice, Jeans, Animal feed, Hydrolases, Fungi, Industrial microbiology

## Abstract

The filamentous *Thermoascus aurantiacus* fungus characterized by its thermophilic nature, is recognized as an exceptional producer of various enzymes with biotechnological applications. This study aimed to explore biotechnological applications using polygalacturonase (PG) derived from the *Thermoascus aurantiacus* PI3S3 strain. PG production was achieved through submerged fermentation and subsequent purification via ion-exchange chromatography and gel filtration methods. The crude extract exhibited a diverse spectrum of enzymatic activities including amylase, cellulase, invertase, pectinase, and xylanase. Notably, it demonstrated the ability to hydrolyze sugarcane bagasse biomass, corn residue, and animal feed. The purified PG had a molecular mass of 36 kDa, with optimal activity observed at pH 4.5 and 70 °C. The activation energy (Ea) was calculated as 0.513 kJ mol^−1^, highlighting activation in the presence of Ca^2+^. Additionally, it displayed apparent *K*_m_, *V*_max_, and *K*_cat_ values of at 0.19 mg mL^−1^, 273.10 U mL^−1^, and 168.52 s^−1^, respectively, for hydrolyzing polygalacturonic acid. This multifunctional PG exhibited activities such as denim biopolishing, apple juice clarification, and demonstrated both endo- and exo-polygalacturonase activities. Furthermore, it displayed versatility by hydrolyzing polygalacturonic acid, carboxymethylcellulose, and xylan. The *T. aurantiacus* PI3S3 multifunctional polygalacturonase showed heightened activity under acidic pH, elevated temperatures, and in the presence of calcium. Its multifunctional nature distinguished it from other PGs, significantly expanding its potential for diverse biotechnological applications.

## Introduction

Biotechnological approaches utilizing microbial enzymes in industrial process provide noteworthy advantages, requiring minimal energy and financial investment. This method contributes to a reduction in environmental impact while utilizing renewable resources^1^. Fungi, among the various enzyme-producing microorganisms, are abundantly found in nature. They exhibit adaptability to diverse substrates, can be cultivated with low costs through supplementation with agro-industrial residues, and are capable of producing significant quantities of extracellular and biodegradable enzymes. These distinctive characteristics position fungi as key players in the production of enzymes with industrial applications, making them a reference in this field^2^.

The thermophilic fungus *Thermoascus aurantiacus* is classified within the phylum Ascomycota, class Eurotiomycetes, order Eurotiales, family Thermoascaceae. It is characterized by its vibrant orange color and elliptical ascospores^3^. Known for its ability to produce a variety of thermostable enzymes, this microorganism demonstrates efficient growth on agro-industrial residues and lignocellulosic biomass. The literature has documented the isolation of 19 strains of *T. aurantiacus*^4,5^. *T. aurantiacus* holds promise for enzyme production, particularly enzymes involved in degrading the lignocellulosic complex of plant cell walls, such as cellulase and xylanase^6^. Notably, the literature lacks information on the production of multifunctional enzymes from wild strains of * T. aurantiacus* that target carbohydrates. Multifunctional enzymes are characterized by containing two or more catalytic modules^7^. Consequently, these enzymes have the potential to simultaneously degrade various carbohydrates present in the plant cell wall.

Pectin constituting the third-largest component of the plant cell wall, is recognized for its intricate, acidic, and high molecular weight structure. This biopolymer is composed of galacturonic acid residues linked by α-1,4 bonds, accompanied by neutral sugars such as arabinoses, galactoses, xyloses, and rhamnoses^8^. The hydrolysis of pectin involves a coordinated action of enzymes including endopolygalacturonase (EC. 3.2.1.15), which randomly cleaves α-1,4 glycosidic bonds within polysaccharide chains. Additionally, exopolygalacturonase type I (EC. 3.2.1.67), specifically targets D-galacturonic acids at non-reducing terminals and exopolygalacturonase type II (EC. 3.2.1.82), releases di-galacturonates from the non-reducing terminal of polygalacturonic acid. These enzymatic activities, among others contribute to the comprehensive hydrolysis of pectin^9^.

Pectinases hold significant industrial relevance, and understanding their biochemical properties is imperative for their effective utilization in pertinent industries. The utilization of pectinases finds application in diverse industries including: textiles, by degumming fibers derived from coconut, jute, flax, ramie and sunn hemp^10–14^; fruit juice processing^15,16^; removal of the mucilage coat from coffee beans^17^; olive oil extraction^18^ and treatment of industrial effluents containing pectic substances^19^. The exploration of novel fungal strains and the comprehension of their pectinase characteristics are imperative to augment their efficiency, with a particular focus on enhancing beverage filtration^20^, and processes improvement without compromising nutritional content, color, or flavor, highlighting the importance of understanding, and optimizing pectinase performance in various industrial applications^21^.

The economic viability and environmental advantages of employing pectinases for the degradation of agro-industrial residues are evident, as this approach helps reduce the accumulation of such residues in nature^22^. In countries like Brazil, a major agricultural producer, there is a notable underutilization of agro-industrial waste, leading to environmental concerns^23^. Enzymes play a crucial role in converting such waste materials, including sugarcane bagasse and forest residues, into valuable products such as second-generation ethanol^24^. Furthermore, enzymes contribute significantly to sustainable practices, ranging from denim treatment^25^ to enhancing animal feed. In the context of animal feed, enzymes improve nutrient availability and digestibility, thereby contributing to increased profitability^26^. These diverse applications underscore the versatility and importance of pectinases in addressing both economic and environmental challenges associated with agro-industrial waste.

Therefore, the primary objective of this study was to produce, purify, and conduct a biochemical characterization of a multifunctional polygalacturonase (PG) enzyme derived from the wild strain *T. aurantiacus* PI3S3. The investigation sought to assess the potential biotechnological applications of this enzyme in areas such as fruit juice clarification, degradation of agro-industrial residues, denim treatment, and improvement of animal feed.

## Results

### The utilization of purified polygalacturonase from *T. aurantiacus* PI3S3 to enhance the clarity of fruit juices

The employment of *T. aurantiacus* PI3S3 polygalacturonase (PG) in the treatment of fruit juices was based on its ability to break down pectin-derived substances within the enzyme, using natural pectin sources such as apple, mango, and orange. The results of the conducted tests are outlined in Supplementary Table S1. The introduction of 5 U mL^−1^ of PG to the juices resulted in an increased release of reducing sugars, measuring 30.4 mg mL^−1^, 13.1 mg mL^−1^, and 16.1 mg mL^−1^ in apple, mango, and orange juices, respectively. Significantly, this addition led to a respective increase in transmittance percentages by 21.2%, 4.2%, and 3.0%, along with a reduction in color of 35.0%, 12.6%, and 28.8%, respectively.

### Hydrolysis of biomass for sugar production

**Figure 1:**
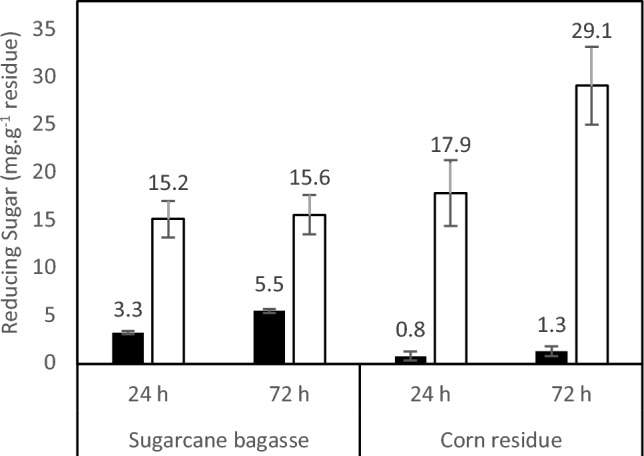
The impact of *T. aurantiacus* PI3S3 crude extract on the breakdown of sugarcane bagasse and corn residue is illustrated by symbols: (filled square) Control; (open square) Treatment. In the assays, 20 mg of each material were exposed to 0.2 mg of the crude extract along with 1.5 mL of 100 mM citrate buffer at pH 5.0. The samples underwent incubation at 50 °C for 24 and 72 h, followed by a 10-min boiling session and centrifugation at 10,000*g* for 10 min. The resulting supernatant was used to determine reducing sugar levels using the method outlined by Miller (1959). The control group involved replacing the crude extract with the same buffer solution.

The crude enzymatic extract from the *T. aurantiacus* PI3S3 fungus exhibited diverse glycosyl hydrolase activities, including amylase (20.86 U mL^−1^), cellulase (10.46 U mL^−1^), invertase (1.65 U mL^−1^), pectinase (29.14 U mL^−1^), and xylanase (41.84 U mL^−1^) (data not displayed). When applied to assess biomass hydrolysis, the enzyme complex demonstrated activity on both tested substrates, sugarcane bagasse and corn residue (Fig. 1). Notably, the highest release of reducing sugars, measuring 29.1 mg g^−1^, was observed from corn residue after 72 h of treatment, compared to 15.6 mg g^−1^ from sugarcane bagasse. Comparing the 24- and 72-h incubation periods for each residue revealed no significant difference in substrate degradation and subsequent sugar release, indicating a minor increase.

### Treatment of animal feed and laboratory-based digestibility evaluations

**Figure 2:**
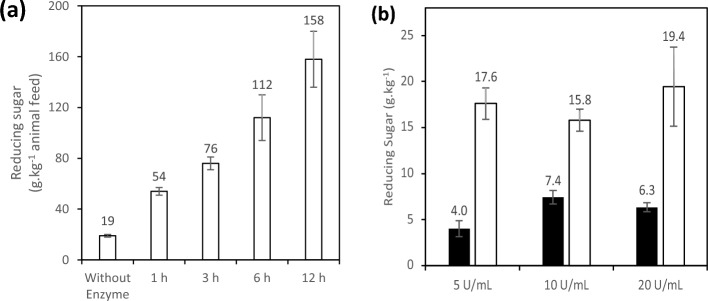
The impact of *T. aurantiacus* PI3S3 crude enzymatic extract on animal feed is depicted in (**a**) and its effect on the liberation of reducing sugars during a digestion simulation is shown in (**b**). Represented by symbols: (filled square) Control; (open square) Treatment. In the digestion simulation conducted in 1.5 mL test tubes, 0.25 g of animal feed was combined with 0.5 mL of either distilled water or the crude extract, at 5, 10, and 20 U mL^−1^ of polygalacturonase. The pH was adjusted to 5.9, and the samples were incubated at 40 °C for 30 min at 200 rpm. Following this, the pH was adjusted to 2.9, and 0.25 mL of 6000 U mL^−1^ pepsin was introduced. The samples were then incubated at 40 °C with orbital shaking at 200 rpm for 45 min. Next, 0.25 mL of 3.7 mg mL^−1^ pancreatin was added, the pH was corrected to 6.1, and the samples were further incubated at 40 °C with orbital shaking at 200 rpm for 2 h. Eventually, the samples were centrifuged at 10,000 rpm for 10 min, and the resultant supernatant was employed to quantify the presence of reducing sugars (Miller 1959).

The experimental trials applying enzymes in animal feed are depicted in Fig. 2a. According to the findings, the animal feed treated with the crude enzymatic extract of *T. aurantiacus* PI3S3 underwent hydrolysis, resulting in the release of 158 g of reducing sugar per kilogram of feed within a 12-h treatment period. Notably, when compared to the control treatment (19 g), the durations of 1, 3, and 6 h led to respective increases in reducing sugar release by 54, 76 and 112 g, respectively.

The liberation of reducing sugar was employed to examine the impact of feed treatment with the crude enzymatic extract in simulated in vitro digestibility trials. Illustrated in Fig. 2b, there was an increase in sugar release within the diets across all tested enzyme concentrations compared to the controls. The results exhibited a surge of 340.0%, 113.5%, and 207.9% in the presence of reducing sugar in the diets treated with 5, 10, and 20 U mL^−1^, respectively. Particularly noteworthy was the highest result observed in the presence of 20 U mL^−1^, accounting for 19.4 g of reducing sugar per kilogram of animal feed.

### Purification of polygalacturonase

**Figure 3:**
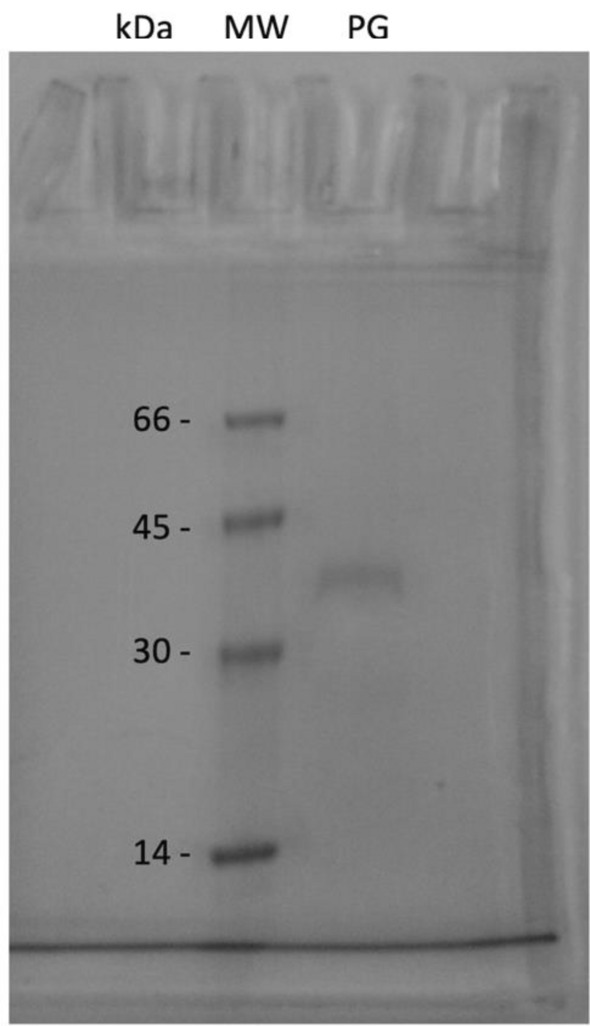
The purified polygalacturonase obtained from *T. aurantiacus* PI3S3 underwent electrophoresis. Represented by symbols: (MW) Molecular weight marker; (PG) Purified polygalacturonase. The enzyme's purity was assessed through electrophoresis using a 10% polyacrylamide gel (SDS–PAGE).

The polygalacturonase (PG) from *T. aurantiacus* PI3S3 underwent a purification process utilizing enzymatic extract in two chromatographic columns. Initially, the extract was subjected to DEAE-Sephadex ion exchange chromatography followed by a Sephadex-G75 gel filtration column. The purification results are outlined in Supplementary Table S2. The crude enzymatic extract displayed a total activity of 5,613 U and 183 mg of protein. The application of this extract in the DEAE-Sephadex ion exchange chromatographic column and subsequently in the Sephadex-G75 gel filtration column resulted in a purification factor of 1.8 times, with a 4% recovery rate, and a specific activity of 57 U mg^−1^. The purification of *T. aurantiacus* PI3S3 PG was confirmed through SDS-PAGE gel analysis, as shown in Fig. 3. An evident single band with an approximate molecular weight of 36 kDa was identified, confirming the effectiveness of the purification process.

### Influence of pH and temperature on polygalacturonase functionality, and determination of activation energy

**Figure 4:**
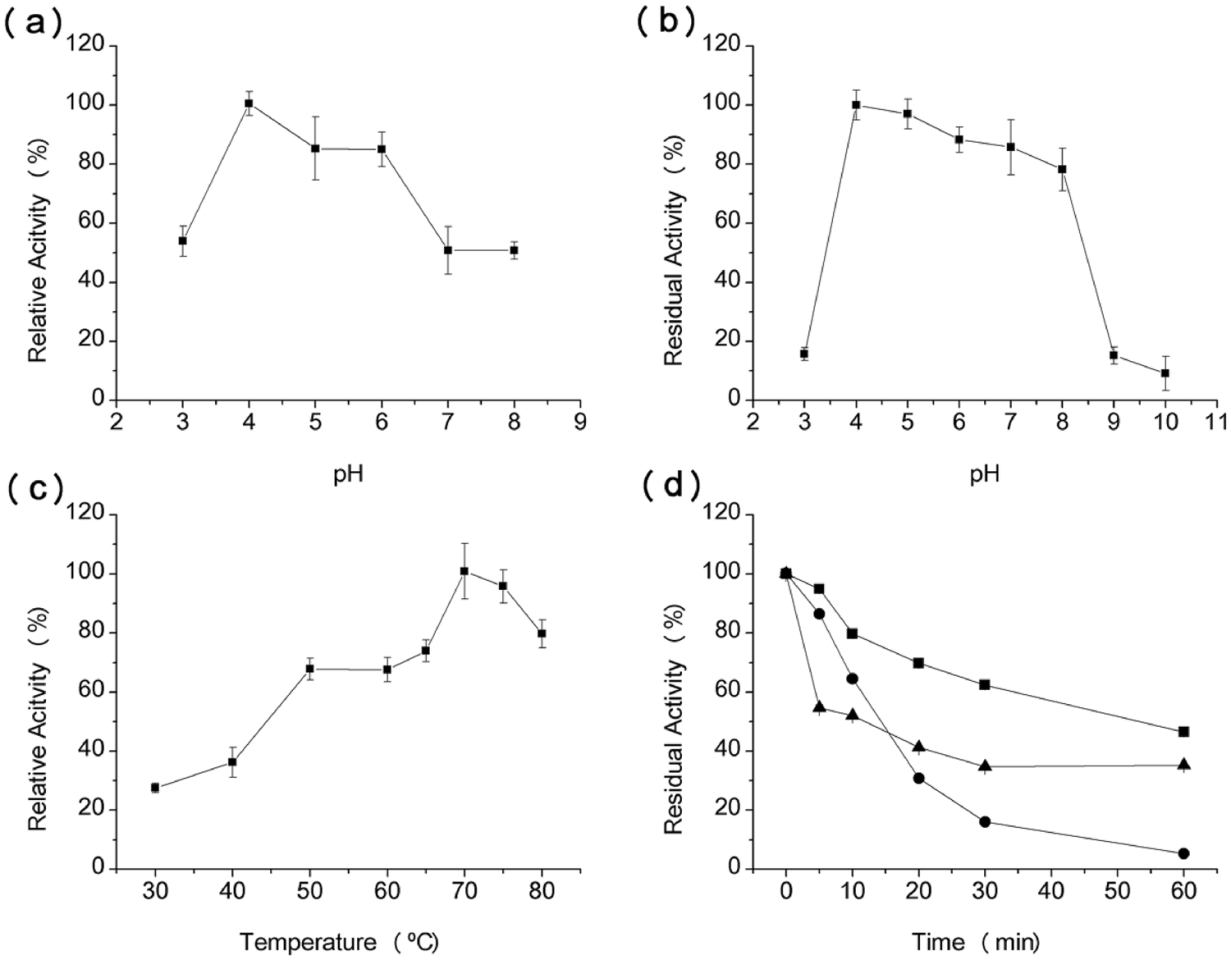
Impact of pH and temperature on both the activity and stability of polygalacturonase: (**a**) determination of optimal pH, (**b**) assessment of pH stability, (**c**) determination of optimal temperature and (**d**) evaluation of temperature stability. Represented by symbols: 50 °C (filled square); 60 °C (filled circle); and 70 °C (filled triangle). To explore the effect of pH on enzymatic activity, the reaction substrate was dissolved in citrate–phosphate buffer (McIlvaine buffer) ranging from pH 3.0–8.0, and in glycine–NaOH buffer at pH 9.0 and 10.0. Subsequently, the residual enzymatic activity was evaluated. pH stability was examined by dissolving the enzyme in the absence of substrate in 100 mM citrate–phosphate buffer (McIlvaine buffer) and NaOH glycine buffer adjusted to the studied pH values, followed by a 24-h incubation at 4 °C. Afterward, the pH was adjusted to 4.0 using sodium acetate buffer (200 mM), and the remaining enzymatic activity was determined. The optimal temperature for polygalacturonase (PG) activity was investigated at various temperatures ranging from 30 to 80 °C. The assessment of thermostability was conducted at 50 °C, 60 °C, and 70 °C, with an incubation time of up to 60 min, followed by an evaluation of residual enzymatic activity.

The impact of pH variation was examined within a range from 3.0 to 10.0, pinpointing the maximum activity of the purified PG at pH 4.0 (Fig. 4A). The enzyme's pH stability was investigated by incubating it at 4 °C across different pH values for 24 h, revealing that the PG maintained robust stability within the pH range of 4.0–8.0 (Fig. 4B), retaining over 80% of its activity.

**Figure 5:**
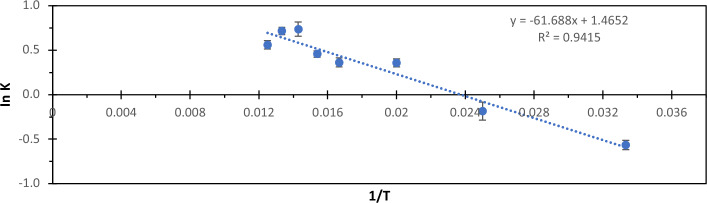
Arrhenius graph used to calculate the activation energy (Ea) of polygalacturonase sourced from *T. aurantiacus* PI3S3. The activation energy (Ea) was derived by applying the Arrhenius equation, where the slope of the semi-logarithmic graph of natural logarithm of the rate constant (lnk) plotted against the reciprocal of temperature (1/T) was utilized for the calculation.

The optimal temperature for PG function was established at 70ºC (Fig. 4C). In thermal stability tests, the enzyme displayed 65% residual activity after 30 min and a half-life (T 1/2) of around 60 min at 50 °C (Fig. 4D). At higher temperatures of 60 °C and 70 °C, the half-life was roughly 15 min. The activation energy for the enzymatic reaction facilitated by PG was calculated at 0.513 kJ mol^−1^ (Fig. 5).

### Impact of metal ions on polygalacturonase functionality

The impact of various ions at concentrations of 1 mM and 10 mM was studied to examine their effect on PG activity. According to the data in Supplementary Table S3, the maximum activity was observed in the presence of 1 mM Ca^2+^, exhibiting a remarkable 316.6% surge when compared to the EDTA control. Additionally, Mg^2+^, Zn^2+^, and Cu^2+^ ions also demonstrated increased activity, showing enhancements of 272.9%, 213.6%, and 242.7%, respectively. However, the presence of Ba^2+^ and Zn^2+^ ions at a concentration of 10 mM entirely inhibited the enzyme activity. Moreover, the Hg^2+^ ion led to a reduction of enzyme activity by 52% and 46.4% at the respective concentrations of 1 mM and 10 mM.

### Polygalacturonase affinity for varied substrates and assessment of kinetic properties

The polygalacturonase extracted from *T. aurantiacus* PI3S3 displayed varying specific activities when exposed to polygalacturonic acid, xylan, and carboxymethylcellulose. As indicated in Table 1, the PG exhibited notable specific activity, particularly in the presence of polygalacturonic acid, displaying a specific activity of 197.17 U mg^−1^ and a *V*_max_ of 273.1 U mL^−1^. Moreover, the enzyme demonstrated activity in the presence of carboxymethylcellulose and xylan, presenting specific activities of 21.72 U mg^−1^ and 15.81 U mg^−1^, along with *V*_max_ values of 209.9 U mL^−1^ and 17.42 U mL^−1^, respectively. These findings suggest that apart from its impact on polygalacturonic acid, the enzyme showcases significant activity in the presence of cellulose compounds, indicating a dual enzymatic functionality.

**Table 1:** The enzyme's specificity for substrates and the kinetic parameters of the polygalacturonase isolated from *T. aurantiacus* PI3S3.

Substrate	Glycosidic bond	Specific activity (U mg^−1^)		*K*_m_ (mg mL^−1^)	*V*_max_ (U mL^−1^)	*K*_cat_ (s^−1^)	*K*_cat_/*K*_m_ (mg^−1^ mL s^−1^)
Polygalacturonic acid	GalA(α1 → 4)GalA	197.17 ± 0.195		0.19	273.10	168.52	886.84
Carboxymethyl celulose	Glc(β1 → 4)Glc	21.72 ± 0.104		61.01	209.90	129.50	2.12
Xylan	Xyl(β1 → 4)Xyl GA(α1 → 2)Xyl	15.81 ± 0.082		8.21	17.42	10.75	1.31

The purified PG was incubated with polygalacturonic acid, xylan and carboxymethylcellulose followed by the enzymatic assay at 70°C pH 4.0, and the kinetic constants (*K*_m_, *V*_max_, *K*_cat_ and catalytic efficiency) of the enzyme were determined using each substrate in concentrations from 1 to 20 mg mL^−1^, according to Michaelis–Menten^23^.

*GalA* galacturonic acid, *Glc* glucose, *Xyl* xylose, *GA* glucuronic acid.

### Examination of breakdown products from polygalacturonic acid

**Figure 6:**
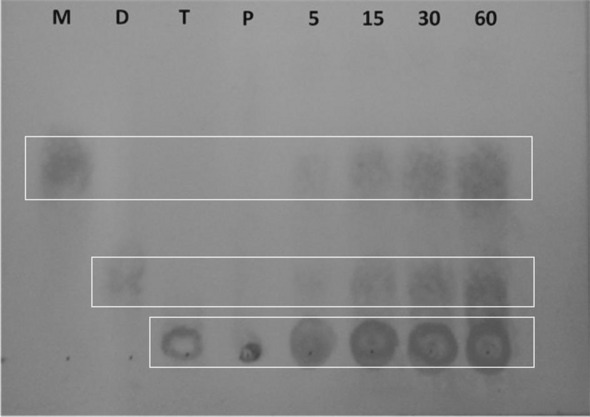
Silica gel thin layer chromatography (TLC) examination of the purified polygalacturonase derived from *T. aurantiacus* PI3S3. Indicated symbols: (M) Monogalacturonic acid 1 mg mL^−1^; (D) Digalacturonic acid 1 mg mL^−1^; (T) Trigalacturonic acid 1 mg mL^−1^; (P) Polygalacturonic acid 1%; (5) 5-min hydrolysis; (15) 15-min hydrolysis; (30) 30-min hydrolysis; (60) 60-min hydrolysis. The experiments involved incubating 25 µL of the pure enzyme with 25 µL of polygalacturonic acid 1% (w/v) in 0.1 M sodium acetate buffer at pH 5.0 at 70 °C for varying durations (ranging from 5 to 60 min). Hydrolysis was stopped by subjecting the aliquots to a temperature of 100 °C for 3 min and the analysis was performed using silica-based thin-layer chromatography (TLC). The mobile phase consisted of butanol, ethanol, and deionized water (in the ratio 5:3:2, v/v/v), and the products were visualized by using a 0.3% (w/v) orcinol solution in a mixture of sulfuric acid and methanol (at 1:9, v/v) after incubation at 100 °C until the chromatographic bands became visible.

The degradation of polygalacturonic acid was evaluated by qualitative analysis, wherein the hydrolysis products were visualized after thin layer chromatography. Three products were identified: monogalacturonic, digalacturonic, and trigalacturonic acids (Fig. 6). The observed activity of the enzyme suggests that *T. aurantiacus* PI3S3 PG engages in both a random (endo-PG) and terminal (exo-PG) mode of action.

### Biofinishing treatment for denim fabric

**Figure 7:**
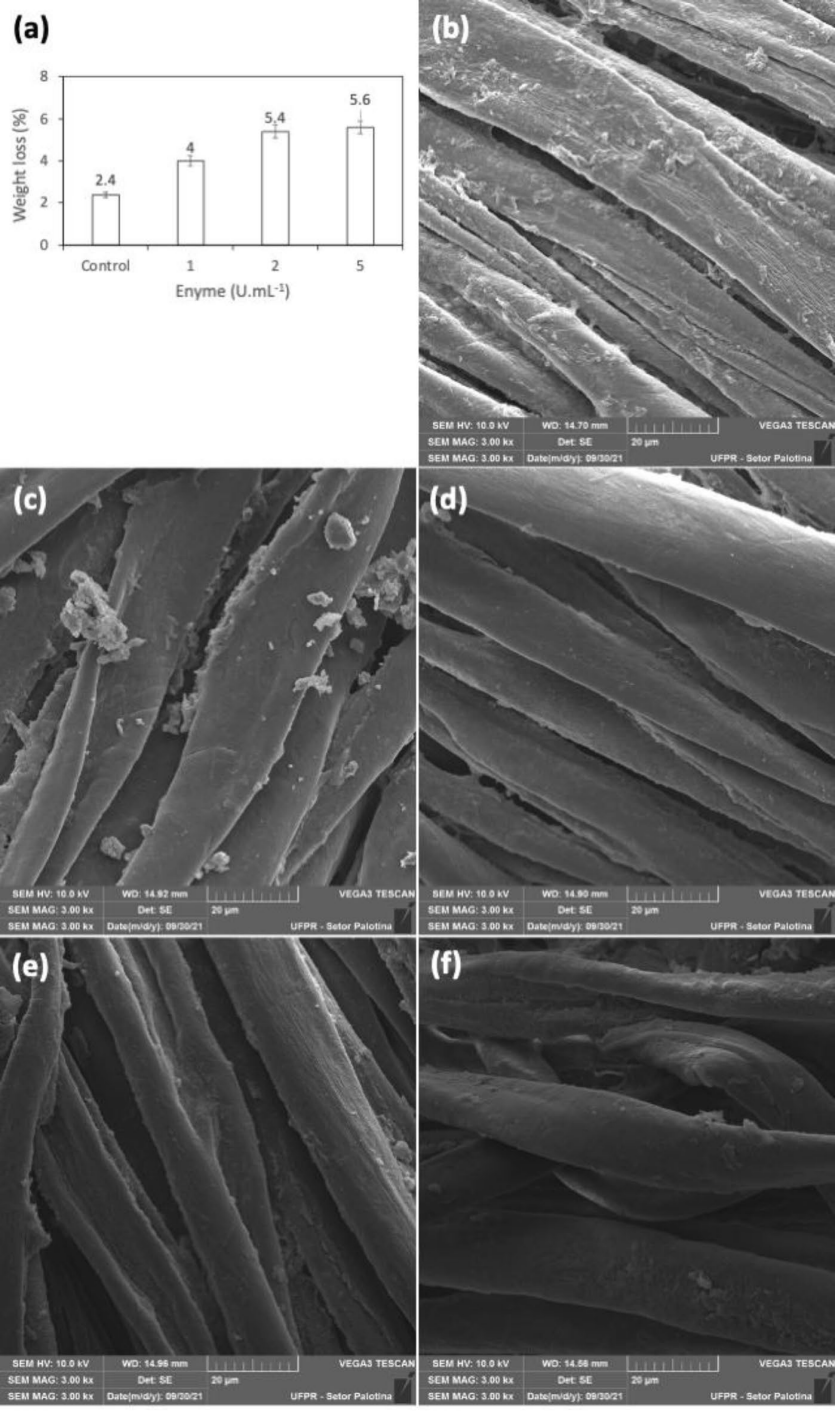
The impact of applying the purified *T. aurantiacus* PI3S3 polygalacturonase on denim jeans fabric. (**a**) Weight loss of jeans according the treatments; scanning electron microscopy analysis: (**b**) control; (**c**) unprocessed denim; (**d**) Denim treated with 1 U mL^−1^; (**e**) denim treated with 3 U mL^−1^; (**f**) denim treated with 5 U mL^−1^. Fabric sections measuring 1 × 1 cm were treated with varying concentrations of the purified polygalacturonase enzyme in sodium citrate buffer at pH 5, and maintained at 70 °C for 12 h without agitation. Subsequently, the mixture was subjected to boiling for 5 min, followed by rinsing with distilled water and air-dried at room temperature. The control treatment was conducted under the same conditions, but without the addition of the enzyme.

The outcomes of the enzymatic treatment on denim fabric were evaluated based on the reduction in weight. Figure 7a depicts the most significant weight loss, observed with treatments at 5 U mL^−1^ (5.6%) and 3 U mL^−1^ (5.4%), indicating a potential for fiber damage. In contrast, the treatment at 1 U mL^−1^ exhibited a weight loss of only 4%.

The impact of tissue biofinishing was assessed via scanning electron microscopy. As shown in Fig. 7d–f, all treatments contributed to denim biofinishing, evident through the reduction in irregularities and protrusions on the fiber surface compared to the control treatment (Fig. 7b) and untreated fabric (Fig. 7c). The treatment at 1 U mL^−1^ (Fig. 7d) displayed more uniform fibers, indicating minimal damage to the fabric structure, in contrast to the treatments at 3 and 5 U mL^−1^ (Fig. 7e,f).

## Discussion

The raw extract from *T. aurantiacus* PI3S3 fungus, obtained from cultures enriched with passion fruit peel, exhibits robust activity of glycosyl hydrolases, including amylase, cellulase, invertase, pectinase, and xylanase. Previous research has highlighted *T. aurantiacus* strains as significant producers of xylanases and cellulases, particularly when cultivated in media enriched with agro-industrial residues^39^.

In the assessments of biomass hydrolysis, a significant level of hydrolysis was observed in the saccharification of both sugarcane bagasse and corn residue. The findings demonstrate a 40% higher release of reducing sugar in the hydrolysis of corn residue compared to the degradation of sugarcane bagasse. Furthermore, in comparison to the control experiment, the hydrolysis of corn residue also surpassed that of sugarcane bagasse. Within 24 h, the corn residue treated with the raw enzymatic extract amplified the release of reducing sugar by 22.4 times, in contrast to the control treatment, while sugarcane bagasse showed an increase of only 4.6 times. This disparity was more pronounced after 72 h of treatment, where the release of reducing sugar from corn residue increased by 22.4 times, while that from sugarcane bagasse increased only by 2.8 times. Nunes et al.^40^ presents promising outcomes regarding the saccharification of agro-industrial residues, underscoring the potential for second-generation ethanol production in Brazil. Consequently, one of the primary challenges still remains the cost-effective production of these enzymes^41^.

The raw enzymatic extract of *T. aurantiacus* PI3S3 was successful in liberating reducing sugars from treated chicken feed at varying concentrations and times, both directly and in simulated in vitro digestibility assays. These outcomes align with previous literature. For instance, Maller et al.^42^ discussed the liberation of substantial phosphate and reducing sugar levels in vitro in diets treated with the enzymatic extract of *Aspergillus japonicus*, comprising phytase, xylanase, cellulase, and amylase. Various enzymes have been shown to have potential beneficial effects on animal feed. Neto et al.^43^ studied the application of *A. niger* URM 7131 tannase for simulating in vitro digestion in monogastric animals, indicating that the enzyme contributed to alleviating the unfavorable effects of tannins. Moreover, in vivo studies have displayed the positive effects of employing enzymes in chicken feed. For instance, Andrade et al.^44^ evaluated the use of enzyme complexes containing xylanase and amylase in a diet primarily based on corn and soybeans, demonstrating an enhancement in nutrient digestibility and broiler performance. Furthermore, Osman et al.^45^ demonstrated that the negative impact of wheat on bone mineralization in laying hens can be mitigated by supplementing the diets with xylanase, β-glucanase, cellulase, and α-amylase.

The purification of *T. aurantiacus* PI3S3 polygalacturonase was achieved through DEAE-Sephadex ion exchange chromatography and Sephadex-G75 gel filtration to isolate the enzyme from the crude extract. Martins et al.^46^ purified a similar enzyme from the *T. aurantiacus* CBMAI-756 strain using Sephadex G-75 gel filtration and SP Sepharose ion exchange chromatography, estimating its molecular weight at 35 kDa, which closely matched our findings (36 kDa) from another strain of the same species. Additionally, Barreto^47^ conducted the purification of polygalacturonase from the *Chrysoporthe cubensis* fungus through ion exchange chromatography and an FPLC (Fast Protein Liquid Chromatography) system utilizing a Sephacryl S-200 column, achieving a purification factor of 3.13. Moreover, Yadav et al.^19^ attained a purification factor of 16.3, with 1.9% recovery and a specific activity of 60.5 U mg^−1^ for *Rhizopus oryzae* PG.

The impact of pH, temperature, and ions on the activity of polygalacturonase isolated from *T. aurantiacus* PI3S3 paralleled findings described in existing literature. For instance, the optimal pH aligns with *Aspergillus niger* MTCC 478's PG, which also operates optimally at pH 4.0 and demonstrates stability within the range of pH 3.0 to 11.0^48^. Similar observations were noted in studies by Damásio et al.^49^ regarding *Paecilomyces variotii*, indicating effective PG activity under acidic pH conditions. Typically, the optimal temperature for PGs described in various studies ranges between 30°C and 60°C, analogous to *Fusarium graminearum's* PG^50^. The thermophilic trait observed in *T. aurantiacus* PI3S3 might explain the discovery of a PG exhibiting a high optimal temperature, as evidenced by Martins et al.^46^ in PG from the *T. aurantiacus* CBMAI-756 strain. In terms of temperature stability, the half-life of *Penicillium viridicatum* at 60°C is extended, lasting 30 min^51^, a contrast to our study's findings, where it was observed to be 15 min under the same temperature. The calculated activation energy presented by the purified polygalacturonase in our study (0.513 kJ mol^−1^) is significantly lower compared to data reported by Mutlu et al.^52^ and Silva et al.^53^, who recorded activation energies of 9.316 kcal mol^−1^ and 9.66 kJ mol^−1^, respectively. However, lower activation energies are advantageous in industrial processes, reducing production costs due to the decreased energy required for the substrate molecule to reach the activated state in the reaction system^53^. Regarding the influence of ions on enzyme activity, divergent results were observed in our research compared to findings by Martins et al.^46^. They indicated that PG from *T. aurantiacus* PI3S3 was significantly activated by 1 and 10 mM concentrations of Mg^2+^ and 1 mM of Zn^2+^, whereas they had observed inhibition by Mg^2+^, Mn^2+^, Zn^2+^, and Hg^2+^ ions, with the exception of Ca^2+^ that enhanced activity.

Understanding the operational mechanisms of the isolated *T. aurantiacus* PI3S3 PG involves evaluating the enzyme's kinetic characteristics. The decision to study the kinetic parameters was driven by the purified enzyme's interaction not only with polygalacturonic acid but also with carboxymethylcellulose and xylan, justifying the investigation of broader substrates beyond polygalacturonic acid. *T. aurantiacus* strains are recognized for producing cellulolytic enzyme complexes and xylanases^54,55^, in addition to polygalacturonases. However, the concurrent breakdown of all three substrates by the PG from the new *T. aurantiacus* PI3S3 strain unveils the enzyme's multifunctional nature, a noteworthy feature with promising biotechnological applications in the food, beverage, and waste treatment sectors. In contrast, Cheng et al.^56^ observed that, when exposed to polygalacturonic acid, citric pectin, apple pectin, carboxymethylcellulose, and xylan, PG from *P. oxalicum* CZ1028 displayed specific activity exclusively for pectic origin substrates, exhibiting no activity in the presence of carboxymethylcellulose and xylan.

The examination of polygalacturonic acid degradation products by the *T. aurantiacus* PI3S3 PG enzyme indicated both random and terminal hydrolytic activity, liberating monogalacturonic, digalacturonic, and trigalacturonic acids simultaneously at the given reaction times. In contrast, Tounsi et al.^31^ observed that PG from *P. occitanis* exhibited a preference for degrading polygalacturonic acid into trigalacturonic acid units, denoting an endo-PG classification. Compared to its *P. occitanis* counterpart, the *T. aurantiacus* PI3S3 PG demonstrates a broader hydrolysis spectrum, capable of concurrent exo- and endo-PG activities.

The outcomes from the enzymatic application in fruit juices demonstrated the effectiveness of PG in clarifying various juices, particularly apple and orange. However, the treatment was notably more efficient in apple juice than in mango and orange juices. Other studies have indicated that PG can also aid in clarifying juices from different fruits, as noted by^30^, who applied this process to pear, banana, and citrus juices.

As per the literature, enzymatic treatments in denim biopolishing typically involve assessing the reduction in weight^57^. Our enzymatic treatments were carried out without agitation, recognizing that the combination of mechanical and enzymatic treatments might cause damage to the fibers, leading to increased weight loss and fiber fragility^58^. This effect could impact fabric strength and yarn quality, thus diminishing its economic value^38^. Our results indicated a decrease in the weight of jeans ranging between 4% and 5.6%. It's worth noting that the ideal weight loss, as reported in literature, falls within the range of 3% to 5%^59^, where higher values suggest excessive tissue degradation. Therefore, the application of *T. aurantiacus* PI3S3 polygalacturonase at 1 U mL^−1^ showed an ideal weight loss of 4%. This observation is supported by scanning electron microscopy, demonstrating the reduction of irregularities and protrusions on the fabric surface while maintaining fiber integrity.

The weight loss data of denim jeans reveal the significant impact of descaling the fabric. Similar results were reported by Bussler et al.^38^ concerning cellulase (CelA) from *C. crescentus*. Their research displayed a weight loss of 2.43%, along with SEM visualization revealing changes in morphology and the removal of impurities without causing fabric fading or damage. Previous literature has detailed how enzymatic treatment can enhance the fabric's quality by increasing strength, softness, and smoothness^57^. Consequently, the *T. aurantiacus* PI3S3 polygalacturonase, as shown in our study, holds the potential to be used in denim biopolishing, reducing costs and environmental impact while enhancing the physical properties and arrangement of fabric fibers, resulting in a clean and smooth surface.

## Conclusion

The crude extract from *T. aurantiacus* PI3S3 demonstrated remarkable effectiveness in enhancing the clarity of apple juice, leading to a significant increase in transmittance and a reduction in color, which is crucial in the sensory evaluation of processed apple juice. Moreover, the extract exhibited notable proficiency in degrading agro-industrial residues, particularly sugarcane bagasse and corn residue, suggesting potential for second-generation ethanol production. It also facilitated the release of reducing sugars from animal feed. Furthermore, the purified polygalacturonase enzyme showed substantial promise for diverse biotechnological applications. Its endo- and exo-polygalacturonase activities covered a wide spectrum of degradation, allowing for the cleavage of polygalacturonic acid glycosidic bonds at various positions within the molecule. The enzyme's unique capability to degrade carboxymethylcellulose and xylan sets it apart from other polygalacturonases. Further investigations will be directed towards the functional and structural analysis of the enzyme, exploring its potential in diverse biotechnological applications, such as its application in textile industry for denim biopolishing.

## Methods

### Fungi variety and cultivation parameters

The recently discovered *T. aurantiacus* PI3S3 strain was previously isolated from samples collected in the subtropical Atlantic Forest region at the public Iguaçu National Park in Paraná, Brazil, with authorization from the manager of the ITAIPU Protected Areas Division. This strain is housed within the Fungi Collection at the Biochemistry Laboratory, part of the Center for Medical and Pharmaceutical Sciences at the Universidade Estadual do Oeste do Paraná (UNIOESTE) in Cascavel, Brazil. To maintain the strain's viability, it underwent regular cultivation on sterile Petri dishes containing 15–20 mL of Potato Dextrose Agar (PDA) medium. Standard maintenance procedures included incubating the strains in an oven at temperatures ranging between 40 and 45 °C for 10 days, followed by refrigeration for a period of up to 30 days.

### Enzyme synthesis

Cultivation occurred in 25 mL Vogel medium^27^, supplemented with 2% passion fruit fiber (w/v), and was initiated by inoculating 1 mL of a spore suspension (NaCl 0.8% and Tween 80 0.05%) into 125 mL Erlenmeyer flasks, employing submerged fermentation. The cultures were then incubated in an orbital shaker at 46 °C for 96 h with agitation set at 120 rpm.

For the extraction of crude enzymatic content, the liquid cultures underwent vacuum filtration through a Büchner funnel and Whatman filter paper No. 1. This process separated the cell-free filtrate from the mycelium. The resulting filtrate was employed for determining polygalacturonase (PG) activity and for subsequent characterization and purification procedures.

### Assessment of enzyme function and quantification of protein levels

Polygalacturonase (PG) activity was assessed usng the 3,5-dinitrosalicylic acid (DNS) reaction, as described by Miller^28^, with polygalacturonic acid serving as the substrate. Calibration of the system was accomplished through a concentration curve of galacturonic acid ranging from 0.1 to 1.0 mg mL^−1^. An enzyme unit was defined as the quantity of enzyme required to liberate 1 µmol of galacturonic acid per minute under the specified test conditions.

Protein quantification followed the Bradford method employing bovine serum albumin as the standard^29^.

### Fruit juices clarification

The influence of polygalacturonase on fruit juice clarification and viscosity reduction was evaluated by applying the enzyme to processed fruit samples. The key parameters investigated included transmittance, color, and flow characteristics of the resulting juice. Adhering to plant guidelines, fruits were procured from the local market, specifically apple (*Malus domestica* var. Gala), mango (*Mangifera indica* L. var. Tommy), and orange (*Citrus sinensis* L. Osbeck var. Pera). The fruits were processed with distilled water and filtered through a 0.1 mm sieve to obtain the juice for testing. Approximately 20 mL of each fruit juice was treated with 50 U (10 mL) of the enzymatic extract from *T. aurantiacus* PI3S3 and incubated at 37 °C for 4 h. Distilled water was added to the juice as a control. Subsequently, the resulting samples were centrifuged for 15 min at 5000 rpm, and the supernatant was analyzed for transmittance (λ = 660 nm), color (λ = 420 nm), reducing sugar content, and pH^30,31^.

### Conversion of biomass into sugars

The assessment of the efficiency in breaking down sugarcane bagasse and corn residue into sugars using the crude enzymatic extract from *T. aurantiacus* PI3S3 followed the methodology adapted from Dudek et al.^32^. Initially, 20 mg of each substrate was treated with 0.2 mg of the crude extract and 1.5 mL of 100 mM citrate buffer at pH 5.0. These samples were incubated at 50 °C for 24 and 72 h, followed by a 10-min boiling phase and subsequent centrifugation at 10,000*g* for 10 min. The resulting supernatant was utilized to quantify the reducing sugar content, employing the procedure described by Miller^28^. The control treatment involved replacing the crude extract with the same buffer.

### Animal feed conditioning

The trials for animal feed treatment were conducted following the methodology adapted from Dudek et al.^32^. This involved incubating 20 mg of animal feed with 0.2 mg of crude extract and 1.5 mL of 100 mM citrate buffer at pH 5.0. These samples were incubated at 70 °C for intervals of 0, 1, 3, 6, and 12 h. Subsequently, they were boiled for 10 min and then centrifuged at 10,000*g* for 10 min. The supernatant obtained was used to quantify the reducing sugar^28^. The control experiment entailed substituting the crude extract with the same buffer.

### Laboratory-based digestibility evaluations

In order to replicate chicken crop and stomach digestibility, we followed the adapted methodology from Zyra et al.^33^. For the crop simulation, 0.25 g of animal feed along with 0.5 mL of either distilled water or crude extract at varying concentrations (5, 10, and 20 U mL^−1^ of PG activity) were placed into 1.5 mL test tubes. The pH was regulated to 5.9 using 20 mM sodium citrate buffer, and the mixture was then incubated at 40 °C for 30 min with an orbital shake set at 200 rpm. Subsequently, the gizzard/stomach digestibility simulation was initiated. The pH was adjusted to 2.9 with 50 mM phosphate buffer, and 0.25 mL of 6000 U mL^−1^ pepsin was added. The samples were then subjected to incubation at 40 °C with an orbital shake at 200 rpm for 45 min. Following this phase, 0.25 mL of 3.7 mg mL^−1^ pancreatin, diluted in 1 M NaHCO_3_, was introduced, and the pH was adjusted to 6.1 using 100 mM sodium citrate buffer. The samples were incubated again at 40 °C with an orbital shake at 200 rpm for 2 h. At the end, the samples underwent centrifugation at 10,000 rpm for 10 min, and the resulting supernatant was utilized to quantify reducing sugars^28^.

### Polygalacturonase purification

Following dialysis of the crude extract in deionized water for roughly 12 h, the resultant extract was balanced with 0.02 M pH 7.5 Tris–HCl buffer. A total of 183 mg of protein from the crude extract was then applied to a DEAE-Sephadex ion exchange chromatographic column (2 × 20 cm) also balanced with 0.02 M pH 7.5 Tris–HCl buffer. Elution was carried out using a sodium chloride gradient (ranging from 0.05 to 1 M). Fractions of 5 mL were collected and assessed for enzymatic activity and protein content. The fractions exhibiting high enzymatic activity were pooled and subjected to dialysis, with all purification steps executed at 4 °C.

Next, the sample was lyophilized, reconstituted in 1 mL of deionized water, and applied to a Sephadex-G75 gel filtration column (60 × 2 cm) balanced with 0.02 M Tris–HCl buffer at pH 7.5. The flow rate was set at 0.05 mL per minute, and fractions of 2 mL were collected for analysis at 280 nm and for enzymatic quantification. The fractions displaying increased PG activity were combined. To assess the enzyme's purity, vertical electrophoresis was performed using a 10% polyacrylamide gel (SDS-PAGE) following the protocol described by Laemmli^34^.

### Influence of pH on the activity and endurance of polygalacturonase

The impact of pH on enzymatic activity was examined by dissolving the reaction substrate (polygalacturonic acid) in McIlvaine buffer (citrate–phosphate buffer) at 100 mM within the pH range of 3.0–8.0 and in glycine–NaOH buffer for pH 9.0 and 10.0. The subsequent step involved assessing the remaining enzymatic activity through Miller's method for measuring reducing sugars^28^. Following this, pH stability was evaluated by dissolving the enzyme in 100 mM McIlvaine buffer (citrate–phosphate buffer) and glycine–NaOH buffer, adjusted to each pH under study. The enzyme-buffer amalgamation without the substrate was incubated at 4 °C for 24 h. Post this period, the pH was regulated to 4.0 using 200 mM pH 4.0 sodium acetate buffer, and the residual enzymatic activity was determined using the methodology outlined by Miller^28^.

### Influence of temperature on polygalacturonase activity

The determination of the optimal temperature for PG activity involved measuring reducing sugars at various temperatures spanning from 30 to 80 °C. For the thermostability investigation, incubation was carried out at 50 °C, 60 °C, and 70 °C for up to 60 min, followed by an assessment of residual enzymatic activity via the method for measuring reducing sugars as detailed by Miller^28^. The enzyme's activation energy (Ea) was derived utilizing the Arrhenius equation^35,36^, calculated from the slope of the semi-log plot of lnk against 1/T:

1 $${\text{lnk}}=-{\text{Ea}}/{\text{RT}}+{\text{lnA}}$$

### Effect of metal ions on polygalacturonase enzymatic function

The influence of ions on PG activity was investigated by immersing the enzyme in a solution containing various ions: Ba^2+^, Ca^2+^, Cu^2+^, K^+^, NH_4_^+^, Hg^2+^, Mg^2+^, Mn^2+^ and Zn^2+^, each at final concentrations of 1 and 10 mM. Subsequently, the enzymatic assay was performed. As a part of experimental control, EDTA was introduced into the sample.

### Kinetic studies

The assessment of substrate specificity was conducted by subjecting polygalacturonase from *T. aurantiacus* PI3S3 to its optimal reaction conditions along with polygalacturonic acid, xylan, and carboxymethylcellulose. This was followed by quantifying the reducing sugars using the Miller method^28^. Additionally, the enzyme's kinetic constants (*K*_m_, *V*_max_,* K*_cat_, and catalytic efficiency) were derived by utilizing concentrations of each substrate ranging from 1 to 20 mg mL^−1^, according to the Michaelis–Menten equation^37^.

### Examination of the breakdown products derived from polygalacturonic acid

The assessment of polygalacturonic acid hydrolysis involved incubating 25 µL of the pure enzyme with 25 µL of 1% polygalacturonic acid (w/v) in 0.1 M sodium acetate buffer at pH 5.0. This reaction took place at the optimal temperature over varying durations, ranging from 5 to 60 min. The hydrolysis process was stopped by subjecting the aliquots to heating at 100 °C for 3 min. Analysis was carried out using thin-layer silica chromatography (TLC). Following this, 10 µL of monogalacturonic acid at a concentration of 1 mg mL^−1^ (w/v), digalacturonic acid at 1 mg mL^−1^ (w/v), and trigalacturonic acid at 1 mg mL^−1^ (w/v) were used as standards. Additionally, 10 µL of 1% polygalacturonic acid and 10 µL of hydrolysis products from different time points were spotted side by side on the plate and dried at room temperature. After development with a solvent system of butanol–ethanol–water (5:3:2, v/v/v), the silica plate was dried at room temperature, sprayed with a solution containing a 0.3% (w/v) orcinol solution in a mix of sulfuric acid and methanol (1:9 v/v) and heated at 100 °C for color development.

### Application of polygalacturonase in the biopolishing of denim fabric (jeans)

The experiments for denim biopolishing were conducted following the adapted methodology from^38^. Pieces measuring 1 × 1 cm, following the manufacturer's specifications (98% cotton and 2% elastane), were utilized. These samples were exposed to purified PG enzyme at varying concentrations (1 U mL^−1^; 3 U mL^−1^; and 5 U mL^−1^) in sodium citrate buffer at pH 5, maintained at 70 °C for 12 h without agitation. Following this, the mixture underwent boiling for 5 min, and the samples were washed with distilled water and air-dried for 48 h at room temperature. A control group was treated under the same conditions, omitting the addition of the enzyme, and the untreated group was the unprocessed crude denim without any treatment. Post-treatment, the denim's morphological structure was examined using scanning electron microscopy (SEM) with a TESCAN® VEGA3 model, capturing micrographs at 5 × magnification in the secondary electron detector module. Additionally, the weight loss of the samples was measured using the following formula:

2 $$\mathrm{Weight loss }\left(\mathrm{\%}\right)=\frac{{\text{W}}1-{\text{W}}2}{{\text{W}}1}x 100$$

### Statistical analysis

All data are the mean of at least three independent experiments. The data were subjected to statistical analysis using OriginPro Learning Edition Software and MSOffice Excel and differences of p < 0.05 were considered to be statistically significant.

### Supplementary Information

Supplementary Tables.

## Data Availability

The datasets generated during and/or analyzed during the current study are available from the corresponding author on reasonable request.
