# Supplementary material for: Purification, biochemical characterization, and biotechnological applications of a multifunctional enzyme from the Thermoascus aurantiacus PI3S3 strain
Source: Sci Rep. 2024 Feb 29;14:5037. doi: 10.1038/s41598-024-55665-7 (PMC10904743; doi:10.1038/s41598-024-55665-7)
Supplement: Supplementary file 1 — Supplementary Tables. [file 41598_2024_55665_MOESM1_ESM.docx]

**Supplementary information**

**Purification, biochemical characterization, and biotechnological applications of a multifunctional enzyme from the *Thermoascus aurantiacus* PI3S3 strain**

Juliane Almeida Battisti^1#^, Giovane Bruno Rocha^1#^, Letícia Mara Rasbold^1^, Vitória Maciel Delai^1^, Monica Sarolli Silva de Mendonça Costa ^2^, Marina Kimiko Kadowaki^1^, José Luis da Conceição Silva^1^, Rita de Cássia Garcia Simão^1^, Thaís Duarte Bifano^1^, Alexandre Maller^1*^

**Table S1** Impact of *T. aurantiacus* PI3S3 crude extract on the clarification of apple, mango, and orange juices, compared to controls without the addition of the crude extract.

|  | **% T_660_** | **% A_420_** | **Reducing sugars**  **(mg.mL^-1^)** | **pH** |
| --- | --- | --- | --- | --- |
| Apple juice | 21.2 | 35.0 | 30.4 | 5.0 |
| Mango juice | 4.2 | 12.6 | 13.1 | 5.0 |
| Orange Juice | 3.0 | 28.8 | 16.1 | 5.0 |

**Table S2** Isolation and purification of polygalacturonase from *T. aurantiacus* PI3S3.

| **Purification step** | **Total activity (Total U)** | **Total protein (mg)** | **Specific activity (U.mg^-1^)** | **Yield  (%)** | **Purification factor** |
| --- | --- | --- | --- | --- | --- |
| Crude extract | 5613 | 183 | 31 | 100 | 1.0 |
| DEAE Sephadex | 1687 | 49 | 34 | 30 | 1.1 |
| Sephadex G75 | 194 | 3.4 | 57 | 4.0 | 1.8 |

The crude extract underwent processing using DEAE-Sephadex ion exchange chromatography, followed by Sephadex-G75 gel filtration. Active fractions were pooled, and their enzyme activity and protein content were measured.

**Table S3** The influence of metal ions on the purified polygalacturonase activity.

| **Metal ions** | **Relative activity (%)** | | | |
| --- | --- | --- | --- | --- |
|  | **1 mM** | | **10 mM** | |
| EDTA* | 100 | ±1.3 | 100 | ±4.0 |
| K^+^ | 117.8 | ±2.3 | 143.3 | ±0.7 |
| NH_4_^+^ | 60.9 | ±5.5 | 153.4 | ±5.4 |
| Mg^2+^ | 272.9 | ±6.3 | 216.5 | ±3.3 |
| Mn^2+^ | 48.7 | ±8.9 | 50.1 | ±5.4 |
| Ca^2+^ | 316.6 | ±4.4 | 107.8 | ±8.2 |
| Ba^2+^ | 186.6 | ±3.8 | N.D. |  |
| Zn^2+^ | 213.6 | ±11.2 | N.D. |  |
| Cu^2+^ | 242.7 | ±4.2 | 8.9 | ±2.5 |
| Hg^2+^ | 52.0 | ±2.5 | 46.4 | ±7.3 |

The purified enzyme was incubated with different ions, such as Hg^2+^, Ba^2+^, Cu^2+^, K^+^, Mg^2+^, Mn^2+^, Ca^2+^, NH_4_^+^ and Zn^2+^, in final concentrations of 1 and 10 mM, followed by the enzymatic assay. The control of the experiment was conducted by adding EDTA to the sample.

*Negative control.

^#^N.D. = not detected.
